# In Vitro neurotoxicity and myotoxicity of Malaysian *Naja sumatrana* and *Naja kaouthia* venoms: Neutralization by monovalent and Neuro Polyvalent Antivenoms from Thailand

**DOI:** 10.1371/journal.pone.0274488

**Published:** 2022-09-12

**Authors:** Nor Asyikin Zukifli, Zalikha Ibrahim, Iekhsan Othman, Ahmad Khaldun Ismail, Janeyuth Chaisakul, Wayne C. Hodgson, Muhamad Rusdi Ahmad Rusmili

**Affiliations:** 1 Department of Basic Medical Sciences, Kulliyyah of Pharmacy, Kuantan Campus, International Islamic University Malaysia, Bandar Indera Mahkota, Kuantan, Malaysia; 2 Department of Pharmaceutical Chemistry, Kulliyyah of Pharmacy, Kuantan Campus, International Islamic University Malaysia, Bandar Indera Mahkota, Kuantan, Malaysia; 3 Jeffrey Cheah School of Medicine and Health Sciences, Monash University Malaysia, Bandar Sunway, Subang Jaya, Malaysia; 4 Department of Emergency Medicine, Universiti Kebangsaan Malaysia Medical Centre, Universiti Kebangsaan Malaysia, Cheras, Malaysia; 5 Department of Pharmacology, Phramongkutklao College of Medicine, Bangkok, Thailand; 6 Monash Venom Group, Department of Pharmacology, Biomedical Discovery Institute, Monash University, Clayton, Victoria, Australia; Weizmann Institute of Science, ISRAEL

## Abstract

*Naja sumatrana* and *Naja kaouthia* are medically important elapids species found in Southeast Asia. Snake bite envenoming caused by these species may lead to morbidity or mortality if not treated with the appropriate antivenom. In this study, the *in vitro* neurotoxic and myotoxic effects *N*. *sumatrana* and *N*. *kaouthia* venoms from Malaysian specimens were assessed and compared. In addition, the neutralizing capability of Cobra Antivenom (CAV), King Cobra Antivenom (KCAV) and Neuro Polyvalent Antivenom (NPAV) from Thailand were compared. Both venoms produced concentration-dependent neurotoxic and myotoxic effects in the chick biventer cervicis nerve-muscle preparation. Based on the time to cause 90% inhibition of twitches (i.e. t_90_) *N*. *kaouthia* venom displayed more potent neurotoxic and myotoxic effects than *N*. *sumatrana* venom. All three of the antivenoms significantly attenuated venom-induced twitch reduction of indirectly stimulated tissues when added prior to venom. When added after *N*. *sumatrana* venom, at the t_90_ time point, CAV and NPAV partially restored the twitch height but has no significant effect on the reduction in twitch height caused by *N*. *kaouthia* venom. The addition of KCAV, at the t_90_ time point, did not reverse the attenuation of indirectly stimulated twitches caused by either venom. In addition, none of the antivenoms, when added prior to venom, prevented attenuation of directly stimulated twitches. Differences in the capability of antivenoms, especially NPAV and CAV, to reverse neurotoxicity and myotoxicity indicate that there is a need to isolate and characterize neurotoxins and myotoxins from Malaysian *N*. *kaouthia* and *N*. *sumatrana* venoms to improve neutralization capability of the antivenoms.

## Introduction

Snake bite envenoming has been recognized as a neglected tropical disease, with an estimated 1.8–2.7 million cases of snake envenoming occurring annually throughout the world [1, 2]. Southeast Asia is a particular area of concern given the number of highly venomous species of snakes found across the geographical range. One of the medically important genera in the region is *Naja spp*. [3–6]. There are two *Naja* species in Malaysia i.e. *Naja sumatrana* and *Naja kaouthia*, which are listed in the Category 1 of medically important venomous snakes [7, 8]. The distribution of *N*. *kaouthia* is relatively wide and covers Indochina, Peninsular Malaysia, North-Eastern India and Southern China [8] whereas the distribution of *N*. *sumatrana* is more restricted in Peninsular Malaysia, Borneo, Sumatra, Thailand and Singapore [9]. Prior to taxonomical revision, *N*. *sumatrana* was previously thought to be identical with *N*. *sputatrix*, the species that was also used for spitting cobra originated from a restricted area in Java, Indonesia [10]. This has created a referencing issue as many earlier studies on *N*. *sputatrix* venom could be conducted by using venom samples from a mixture of Southeast Asian spitting cobra species [11].

Bites from *N*. *sumatran*a or *N*. *kaouthia* an result in local or systemic envenoming [12]. Local envenoming may present as localized pain, progressive oedema and expanding necrosis from the bitten area, without neurotoxicity and cardiotoxicity [13–16]. Venom ophthalmia is a unique outcome from local envenoming by spitting cobra where sprayed venom enters the eyes and causes severe pain, blurred vision, and ocular damage [17]. Important major effects caused by systemic envenoming by both species are neurotoxicity, involving parasympathetic and somatic muscle nervous systems, and cardiotoxicity [13, 18, 19]. Systemic and severe local envenoming caused by *N*. *sumatrana* and *N*. *kaouthia* are treated in Malaysia using either imported Thai Red Cross Cobra Antivenom (CAV) or Neuro Polyvalent Antivenom (NPAV) [13]. NPAV is raised against Malayan krait (*Bungarus candidus*), banded krait (*Bungarus fasciatus*), monocled cobra (*Naja kouthia*) and king cobra (*Ophiophagus hannah*) [20, 21]. The effectiveness and cross neutralization of Thai Red Cross CAV and NPAV have been separately shown in previous in vivo studies [20–23]. There is limited information on comparing the efficacy of Thai CAV and NPAV, especially in in vitro preparation. *N*. *kaouthia* and *N*.*sumatrana* could be misidentified as *Ophiophagus hannah* (king cobra) by untrained personnel due to some of their nearly similar physical look, hooding behavior and signs and symptoms of systemic envenoming in the area where both are endemic. Thai Red Cross King Cobra Antivenom (KCAV) is the only monovalent antivenom used for treating *O*.*hannah* envenoming and it may be wrongly used if the envenoming snake was misidentified. However, the cross-neutralization capability of KCAV against *N*.*sumatrana* and *N*.*kaouthia* venom has never been reported. This study compared the neurotoxicity and myotoxicity of venoms of Malaysian *N*. *kaouthia* and *N*. *sumatrana*, and neutralization with Thai Red Cross antivenoms.

## Materials and methods

### Crude venom collection

Pooled Malaysian *Naja sumatrana* and *Naja kaouthia* venoms were gifts from Mr. Zainuddin Ismail (Perlis, Northwest Peninsular Malaysia). The venom samples were obtained by milking 5 snakes for each species. The snakes were originated from Northwest of Peninsular Malaysia and were milked by positioning the fangs on a plastic container, with a parafilm cover, while the venom glands were externally massaged. Fresh crude venoms were transported on ice to the laboratory at the International Islamic University Malaysia and subsequently frozen at -80°C before being freeze-dried. Freeze-dried venom samples were weighted, labelled, and stored at -20°C prior to use. When required, the venoms were weighed and dissolved in double distilled water. Research permit for venom collection was obtained from the Department of Wildlife and National Parks (Peninsular Malaysia) (Permit No: HQ-00067-15-70).

### Chemical and drugs

The following were purchased from Sigma Aldrich (St. Louis, MO, USA): acetylcholine (ACh), carbamylcholine chloride (CCh), and d-tubocurarine. The following were purchased from Queen Saovabha Memorial Institute, Thai Red Cross Society Bangkok, Thailand: Cobra Antivenom (CAV, Lot no: NK 00316), Neuro Polyvalent antivenom (NPAV, Lot no: NP 00515) and King Cobra antivenom (KCAV, Lot no: LH 00116). The following were purchased from Fisher Scientific: glucose, calcium chloride (CaCl_2_), potassium di-hydrogen phosphate (KHPO_4_), and sodium hydrogen carbonate (NaHCO_3_). All chemicals were dissolved or diluted in double distilled water for experiments.

### Animal ethics and care

Male chicks (4–10 days old) were purchased from a local poultry hatchery (Terengganu, Malaysia) and kept in a well-lit cage with access to food and drinking water *ad libitum*. Approvals for animal use and experimental procedures were obtained from the IIUM Animal Ethics Care and Use Committee, International Islamic University Malaysia (Animal ethic approval ID:IIUM/IACUC-2019(13)).

#### Sodium Dodecyl Sulphate Polyacrylamide Gel Electrophoresis (SDS-PAGE)

SDS-PAGE was conducted in 10% polyacrylamide gel by using the method that has been previously described [28]. *N*. *sumatrana* and *N*. *kaouthia* venoms were dissolved in double distilled water and prepared in non-reducing and reducing sample buffers. The 2x Laemli SDS-PAGE buffer (Bio- Rad, Hercules, USA) was used as non-reducing sample buffer and the reducing sample buffer was prepared by mixing 50 μL of β-mercaptoethanol (Bio- Rad, Hercules, USA) per 950 μL of 2x Laemli SDS-PAGE sample buffer (Bio- Rad, Hercules, USA). The venoms (10 μg) prepared in reducing and non-reducing sample buffers were loaded and electrophoresed for 30 min at 90V and then at 120V for 90 min. The gel was then stained using silver staining. Precision Plus Protein Standard (Bio- Rad) was used in the gel for molecular weight protein markers. The gel was scanned using the Fusin FX Imaging System (Vilber Lourmat, Marne- la-Vallée Cedex 1, France). The image was analyses by Image J using the following parameters; vertical scale factor: 1.0, horizontal scale factor 1.0 and invert peaks. The intensity of the peaks was determined by densitogram generated from the software.

### Western-Blotting

Reduced *N*. *sumatrana* and *N*. *kaouthia* venoms (10 μg) were loaded and electrophoresed in 10% separating SDS-PAGE gel with 5% stacking gel at 90V for 30 min and then 120V for 90 min. The venom was transferred onto polyvinylidene difluoride (PVDF) membrane (Bio- Rad) by semi-dry electroblotting (Bio- Rad, Hercules, CA, USA) at 25V and 1.0A for 30 min. The membrane was the blocked in Tris- buffered saline with 1% Tween 20 (TBST) buffer (20mM Tris, 0.5 M NaCl and 0.5% Tween-20) supplemented with 5% skim milk for 1 h at room temperature to prevent non-specific binding. The primary antibodies i.e., antivenoms were diluted 1:500 fold in TBST with 5% skim milk, were then added onto the membrane before incubated overnight at 4°C. The membrane was then washed three times for 15 min with TBST buffer. Secondary antibody (i.e., goat anti-horse IgG secondary antibody conjugated with horseradish peroxidase (Abcam, Cambridge, UK) diluted in 1:5000 with TBST buffer with 5% skim milk) was added to the membrane and left for 1 h at room temperature. The membrane was then washed three times for 10 min and the membrane incubated with ECL Substrate Kit for 2 min (Abcam, Cambridge, UK). After the incubation, the excess ECL reagent was removed and the membrane were scanned using Fusin FX Imaging System (Vilber Lourmat, Marne- la-—Vallée Cedex 1, France). The blot image was analyzed by similar parameters as in SDS-PAGE image.

### Chick biventer cervicis nerve-muscle preparation

#### Indirect stimulated preparation for neurotoxicity assay

The experiments were conducted using a method previously described with slight modification [28, 29, 31]. Briefly, chicks (7–14 days old) were euthanized by CO_2_ inhalation before the biventer muscles were dissected and mounted in 25 mL organ baths, aerated with carbogen (5% CO_2_ and 95% O_2_). The organ baths were filled with physiological salt solution of the following composition: NaCl, 118.4 mM; KCl, 4.7 mM; MgSO_4_, 1.2 mM KH_2_PO_4_, 1.2 mM; CaCl_2_, 2.5 mM; NaHCO_3_, 25 mM and glucose, 11.1 mM. The tissues were stimulated via a ring electrode every 1 s with pulses of 0.2 ms duration at a supramaximal voltage using a 82415IS square wave stimulator (Lafayette, USA). Indirect stimulation of the tissues was confirmed by the abolishment of twitched by d-tubocurarine (10 μM). The time required to achieve 90% reduction of original indirect twitches height after venom was added (t_90_) was used to determine the potency of neurotoxicity of the venoms. In the absence of electrical stimulation, responses to acetylcholine (ACh; 1 mM for 30 s), carbachol (CCh; 20 μM for 60 s) and KCl (40 mM for 30 s) were obtained. Responses were recorded on a PowerLab system (ADInstruments, Bella Vista, NSW, Australia). For antivenom preincubation studies, antivenom at the recommended titer by the manufacturer i.e. 1 ml antivenom per 0.6 mg venom for Neuro Polyvalent Antivenom (NPAV) and Cobra Antivenom (CAV), and 1 ml antivenom per 0.8 mg venom for King Cobra Antivenom (KCAV), was added into the organ bath 15 min prior to the addition of venom. For antivenom reversal studies, antivenom was added at the t_90_ time point. Neurotoxicity was determined by a reduction in the height of indirect-twitches and contractile responses to ACh and CCh.

#### Directly stimulated preparation for myotoxicity assay

The tissues used for the assay were prepared as per indirectly stimulated preparation except the tissues were stimulated every 1 s with pulses of 2 ms duration at a supramaximal voltage using 82415IS square wave stimulator (Lafayette, USA) attached to silver ring electrodes. D-tubocurarine (10 μM) was added to the organ bath and remained in the organ bath for the duration of the experiment to inhibit nerve function. In the absence of electrical stimulation, responses to KCl (40 mM for 30 s) were obtained. Responses were recorded on a PowerLab (ADInstruments, Bella Vista, NSW, Australia). For antivenom studies, antivenom at the recommended titer as in antivenom reversal study, was added into the organ bath 15 min prior to the addition of venom. Myotoxicity was determined by a reduction in the height of directly stimulated twitches, an increase in baseline of the tissue and a reduction of the contractile response to exogeneous KCl.

### Data analysis

The relative mobility value (R_f_) for peaks in densitogram for SDS-PAGE and western blot was manually calculated by dividing the migration distance of the band for the respective peaks with the maximum distance of dye front. Intensity value was obtained from densitogram using ImageJ software [24]. Changes in twitch height are expressed as a percentage of the average change of the twitch height after addition of venom over the original twitch height i.e., prior to the addition of venom. The twitch height response for 5 sec was selected and averaged for each time point. Changes in the magnitude of contractile responses to exogenous agonists are expressed as a percentage of the original response to the agonist prior to the addition of venom. Data for twitch height and contractile response changes are presented in the form of mean ± SEM. The experiments were repeated at least 3 times as technical replicates unless mentioned otherwise. Data were analyzed by using a one-way analysis of variance (ANOVA) followed by Bonferroni`s comparison test (GraphPad Prism 6, La Jolla, CA, USA).

## Results

### SDS-PAGE

**Fig 1:**
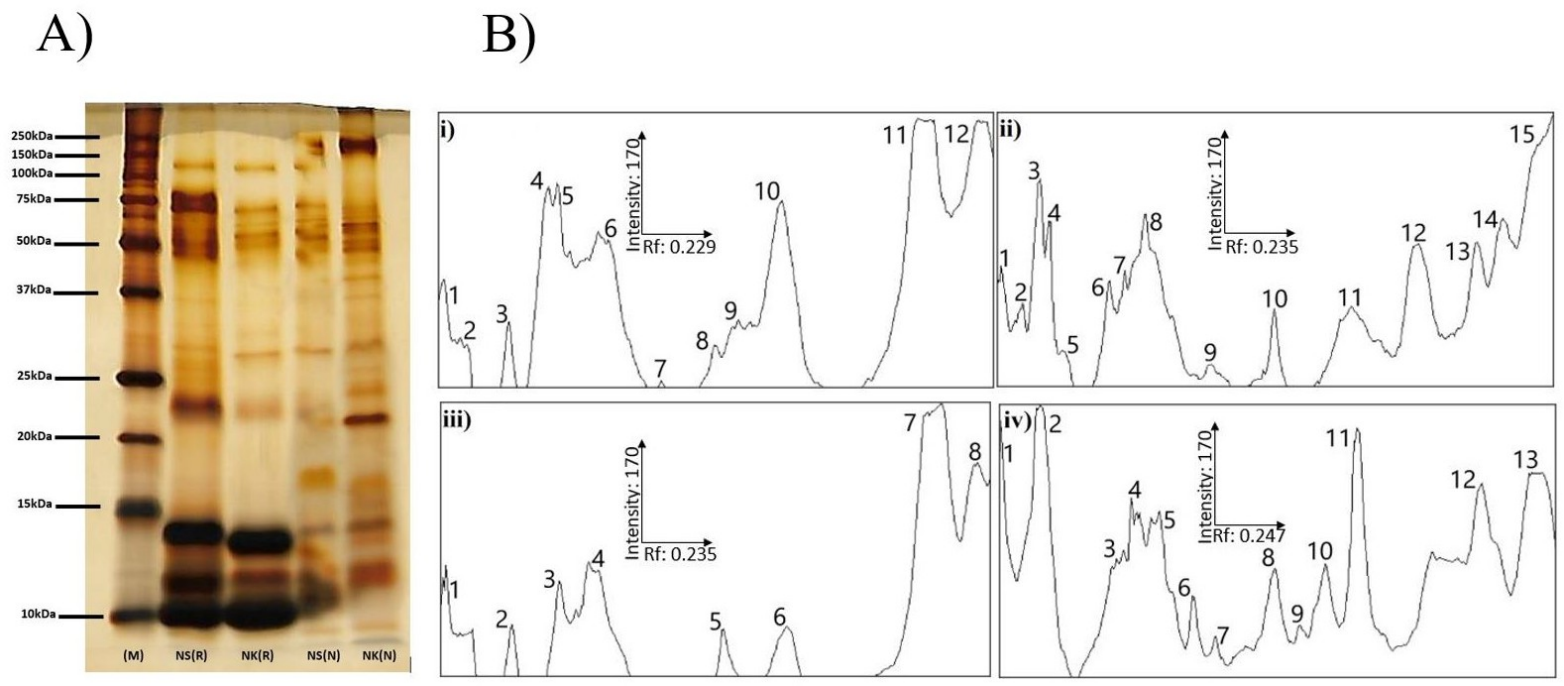
SDS-PAGE and densitogram of venoms in a 10% gel. Venoms were treated in reducing and non-reducing buffer prior to loading before electrophoresed and the gel was stained using silver staining (A). M indicates the protein marker lane, NS indicates *N*. *sumatrana* venom, NK indicates *N*. *kaouthia* venom, (R) indicates venoms treated with reducing sample buffer, (N) indicates venoms treated with non- reducing sample buffer, (i) indicates reduced Malaysian *N*. *sumatrana* venom, (ii) indicates non-reduced Malaysian *N*.*sumatrana* venom, (iii) indicates reduced Malaysian *N*. *kaouthia* venom, (iv) indicates non-reduced Malaysian *N*. *kaouthia* venom. Densitograms were generated using ImageJ software and the relative mobility for protein band (R_f_) corresponding to the peak was manually calculated.

SDS- PAGE analysis of *N*. *sumatrana* and *N*. *kaouthia* venoms shows that there were differences in intensity and pattern of protein bands under non-reduced and reduced conditions (Fig 1A). SDS-PAGE densitogram for the *N*. *sumatrana* venom showed 15 peaks in non-reduced venom and 12 peaks in reduced venom, whereas for *N*. *kaouthia* venom, 13 peaks were detected in reduced venom and 8 peaks were detected in non-reduced venom (Fig 1B).

### Western blotting

**Fig 2:**
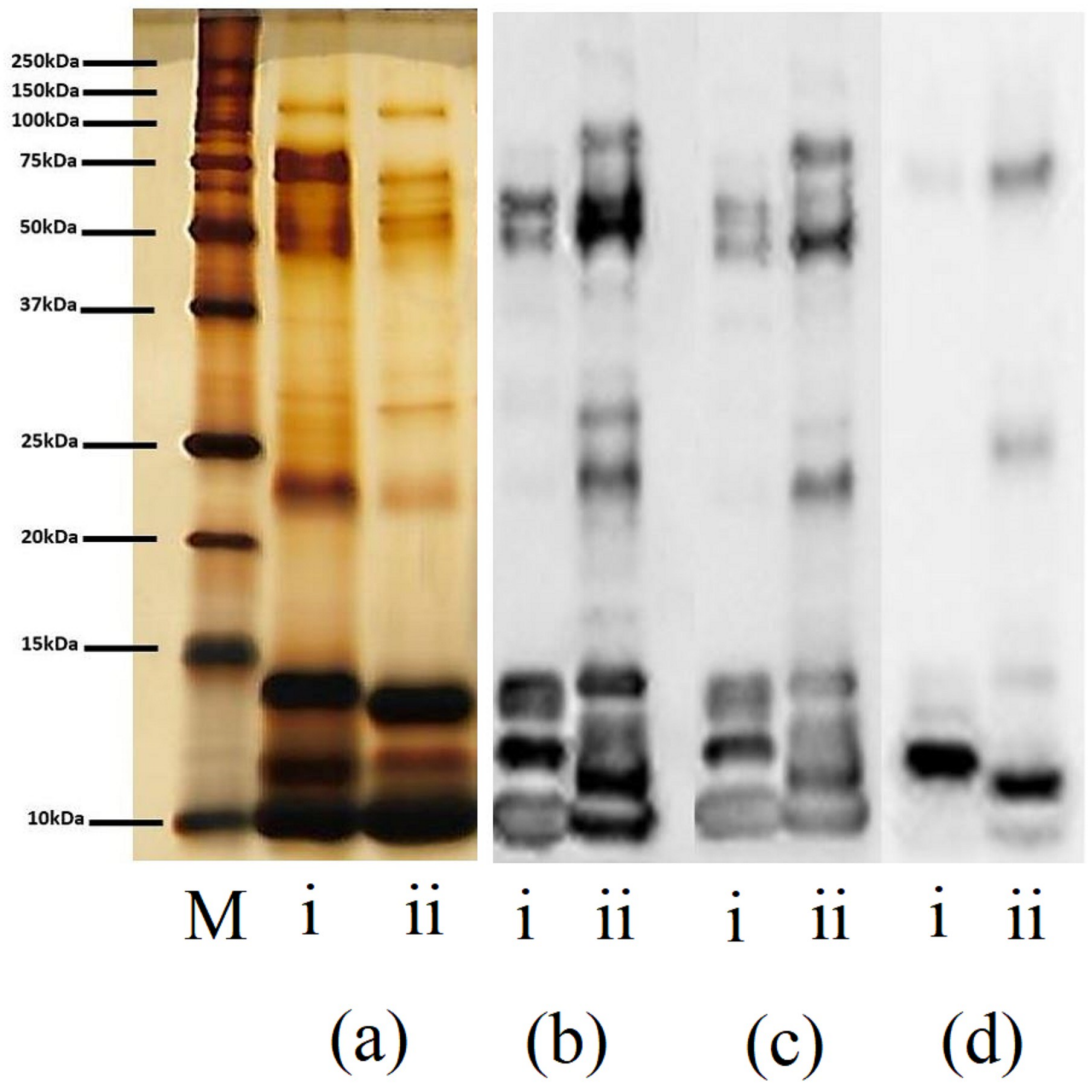
Comparison of SDS-PAGE and western blot profiling of reduced venoms. (i) *N*. *sumatrana* and: (ii) *N*. *kaouthia venoms* (10 μg) were separated in a 10% gel, blotted into a PVDF membrane and visualized using a chemiluminescence substrate. Panel (A) SDS- PAGE profile of venoms. Panel (B-D) are blot membrane that were incubated with Thai Neuro Polyvalent Antivenom, Thai Monocled Cobra Antivenom, and Thai King Cobra Antivenom, respectively. M is lane for protein molecular weight marker.

**Fig 3:**
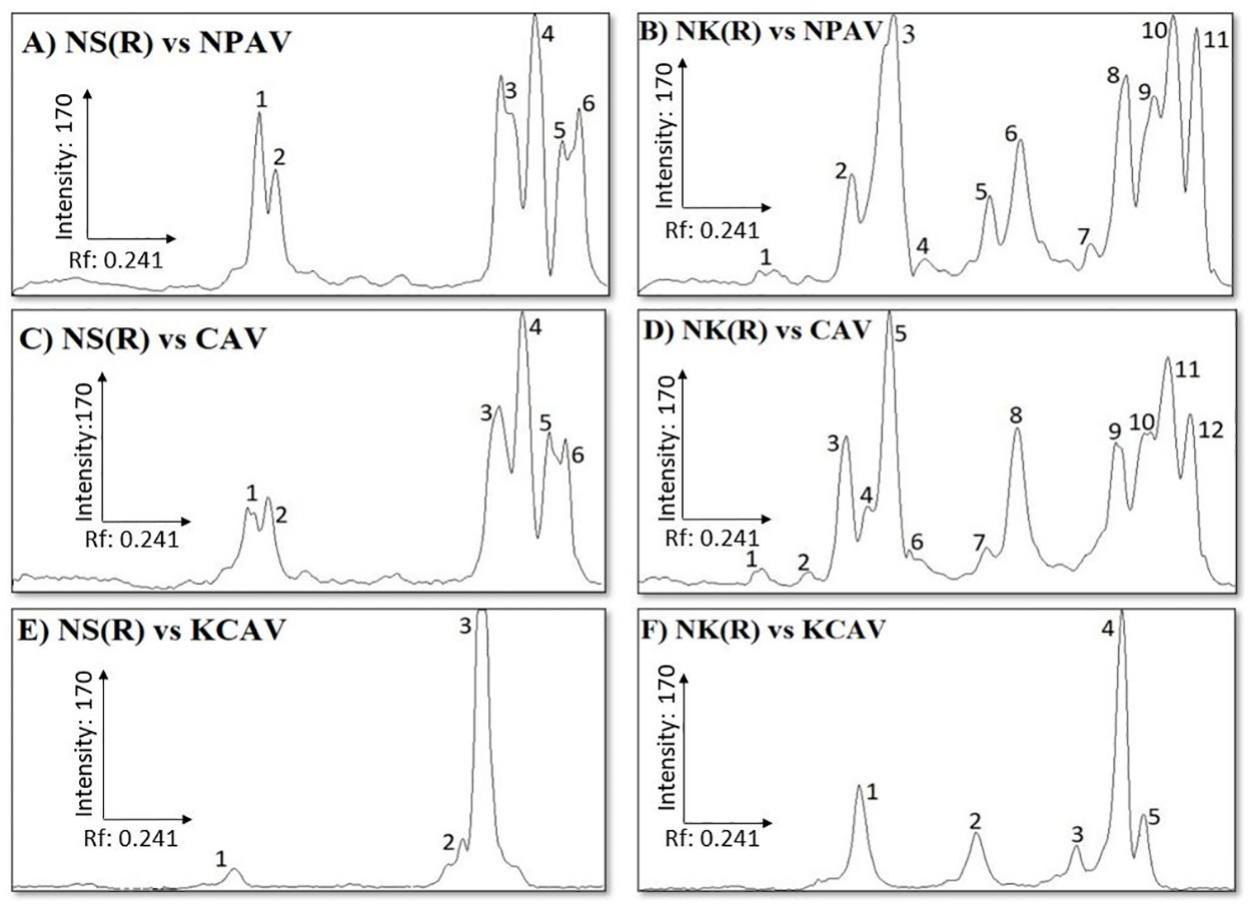
Densitogram for western blot of Malaysian *Naja sumatrana* and *Naja kaouthia* venoms. Panels (A-C) are desnsitograms for membrane blot of *N*. *sumatrana* venom that were incubated with Neuro Polyvalent Antivenom (NPAV), Monocled Cobra Antivenom (CAV) and King Cobra Antivenom (KCAV),respectively. Panels (D-E) are desnsitograms for membrane blot of *N*. *kaouthia* venom that were incubated with Neuro Polyvalent Antivenom (NPAV), Monocled Cobra Antivenom (CAV) and King Cobra Antivenom (KCAV), respectively NS(R) indicates reduced *N*. *sumatrana* venom and NK(R) indicates reduced *N*. *kaouthia* venom. Densitograms were generated using ImageJ software and the relative mobility for protein band (R_f_) corresponding to the peak was manually calculated.

Immunoreactivity profile of blots of gel loaded with reduced *N*. *sumatrana* and *N*. *kaouthia* venoms incubated with CAV, NPAV or KCAV showed differences in their profile (Fig 2). The majority of the protein bands in *N*. *sumatrana* venom that reacted with all three antivenoms in the blots were within the 10–15 kDa and 37–50 kDa ranges, whereas for *N*. *kaouthia* venom, the detected protein bands ranged from 10–75 kDa (Fig 2B–2D). Densitogram of reduced *N*. *sumatrana* blots that were incubated with NPAV and CAV showed 6 peaks (Fig 3A and 3B) compared to 12 in densitogram of SDS-PAGE loaded with reduced *N*. *sumatrana* venom (Fig 1A). Densitogram of reduced *N*. *kaouthia* blots that were incubated with NPAV and CAV showed nearly similar number of protein bands, 11 and 12, respectively (Fig 3D and 3E) compared to 8 in SDS-PAGE densitogram (Fig 1C). Densitogram for *N*. *sumatrana* and *N*. *kaouthia* blots that were incubated with KCAV showed 3 and 5 peaks, respectively (Fig 3C and 3F).

### Neurotoxic effect of *N*.*sumatrana* and *N*.*kaouthia* venoms in indirectly stimulated tissue preparation

**Fig 4:**
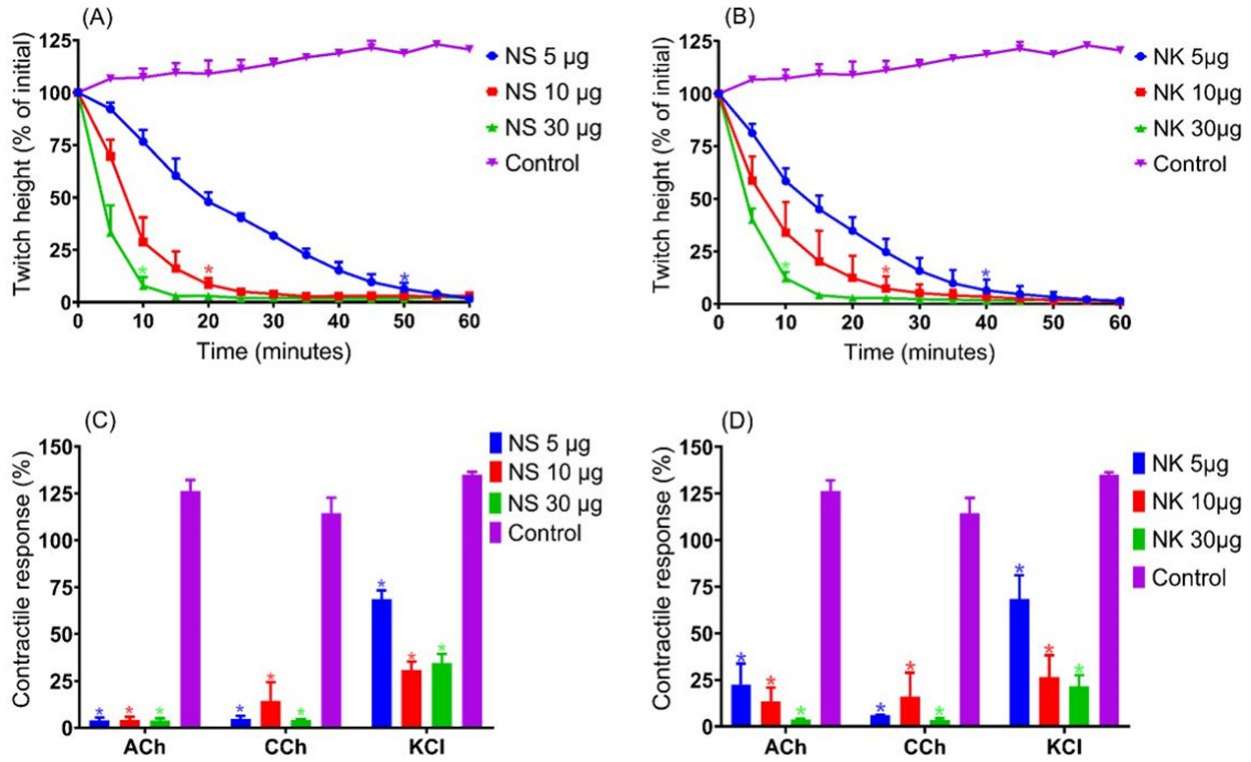
Neurotoxic effects of Malaysian *N*. *sumatrana* venom and *N*. *kaouthia* venom in indirectly stimulated twitches of the chick biventer cervicis nerve-muscle preparation. Panels (A) and (B) indicate change of indirectly stimulated twitch height in tissues exposed in different concentration (5–30 μg/ml) of *N*. *sumatrana* and *N*. *kaouthia* venoms, respectively. Panels (C) and (D) indicate change in the contractile response to exogenous agonist in tissue exposed to different concentration (5–30 μg/ml) of *N*. *sumatrana* and *N*. *kaouthia* venoms, respectively. NS indicates *N*.*sumatrana* venom and NK indicates *N*.*kaouthia* venom * *p* < 0.05, significantly different from control (distilled water) (n = 3, one-way ANOVA).

**Fig 5:**
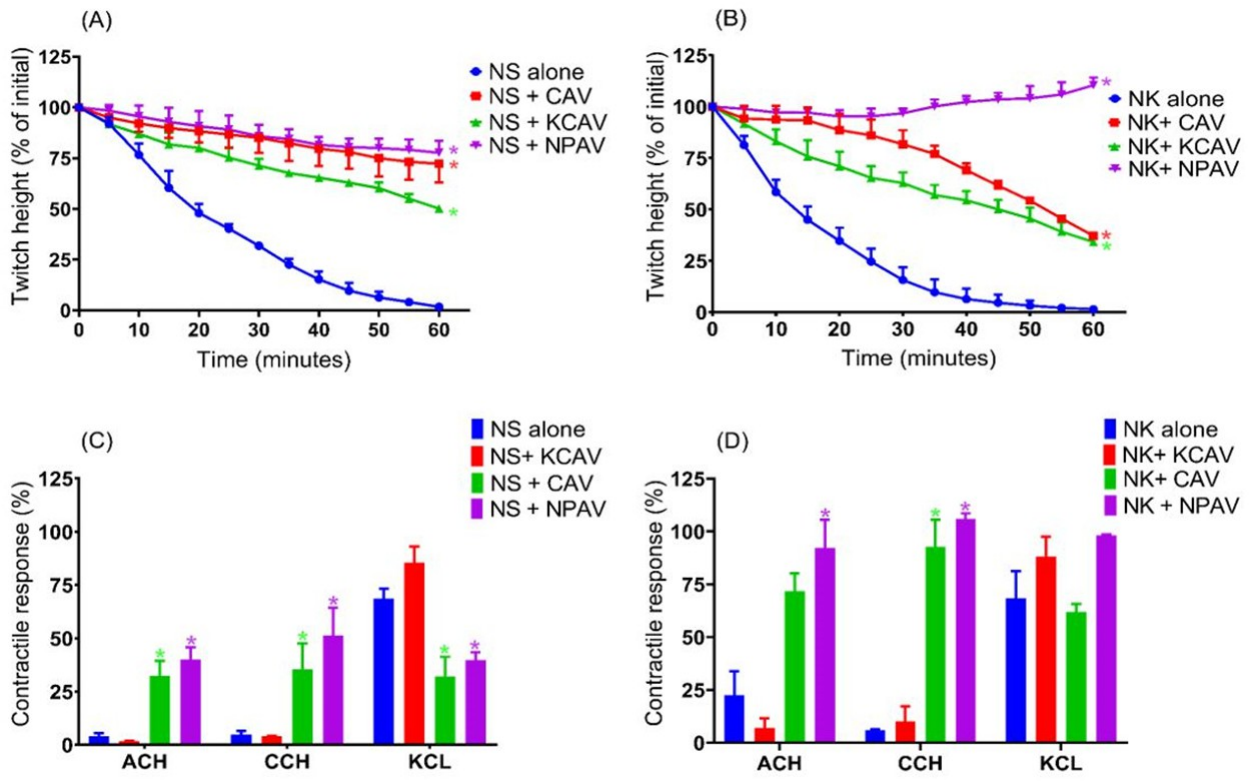
Effect of pre-incubation of antivenoms to indirectly stimulated twitches and contractile responses to exogenous agonists in tissue exposed to Malaysian *N*.*sumatrana* and *N*.*kaouthia*. Panels (A) and (B) indicate change in twitch height for tissues that were pre-incubated with recommended titer of Cobra Antivenom (CAV), Neuro Polyvalent Antivenom (NPAV) and King Cobra Antivenom (KCAV) and exposed to *N*.*sumatrana* and *N*.*kaouthia* venoms (5 μg/ml), respectively. Panels (C) and (D) indicate contractile responses to exogenous agonists in tissues that were pre-incubated with antivenoms after exposure to *N*.*sumatrana* and *N*.*kaouthia* venoms (5 μg/ml), respectively. NS indicates *N*.*sumatrana* venom and NK indicates *N*.*kaouthia* venom. * *p* < 0.05, significantly different than the respective venom alone (n = 3, one-way ANOVA).

Both *N*. *sumatrana* and *N*. *kaouthia* venoms (5, 10 and 30 μg/ml) caused a concentration-dependent reduction of indirectly stimulated twitches in the chick biventer cervicis nerve-muscle preparation (Fig 4A and 4B). The time required for the initial twitches to be reduced by 90% (t_90_) for *N*. *sumatrana* venom at different concentration was: 30 μg/ml, 10 ± 4 min; 10 μg/ml, 20 ± 3 min; 5 μg/ml, 50 ± 2 min and for *N*. *kaouthia* venom was; 30 μg/ml, 10 ± 3 min; 10 μg/ml, 28 ± 4 min; 5 μg/ml, 42 ± 5 min (n = 3; one-way ANOVA, *p* < 0.05). Both venoms markedly attenuated contractile responses to exogenous acetylcholine (ACh), carbachol (CCh) and potassium chloride (KCl) response (Fig 5A and 5B).

### Neurotoxicity prevention by antivenom pre-incubation

Pre-incubation of CAV, KCAV or NPAV prior to the addition of *N*. *sumatrana* or *N*. *kaouthia* venoms (5 μg/ml) showed that all three antivenoms significantly prolonged the time taken to abolish indirect twitches compared with venom alone (Fig 5A and 5B). NPAV significantly prevented the twitch inhibition of both venoms. Tissues that were pre-incubated with NPAV retained 77.6 ± 5.8% of twitch height when exposed to *N*. *sumatrana* venom and no reduction in twitch height was seen in tissues exposed to *N*. *kaouthia* venom at 60 min. CAV and KCAV significantly prolonged the time taken to attenuate twitches following the addition of *N*. *kaouthia* venom (Fig 5B). Tissues that were pre-incubated with CAV and KCAV prior exposure to *N*. *kaouthia* retained 37.2 ± 1.3% and 34.2 ± 4.4% of initial twitch height at 60 min, respectively. Whereas for *N*. *sumatrana* venom, tissue that were pre-incubated with CAV and KCAV retained approximately 72.2 ± 9.2% and 50.1 ± 1.4% of initial twitch response, respectively (Fig 5A). Preincubation with CAV or NPAV prevented inhibition of contractile response to all exogenous agonists in tissues exposed to *N*. *sumatrana* or *N*. *kaouthia* venoms (Fig 5C and 5D).

### Neurotoxicity reversal by antivenom addition at t_90_

**Fig 6:**
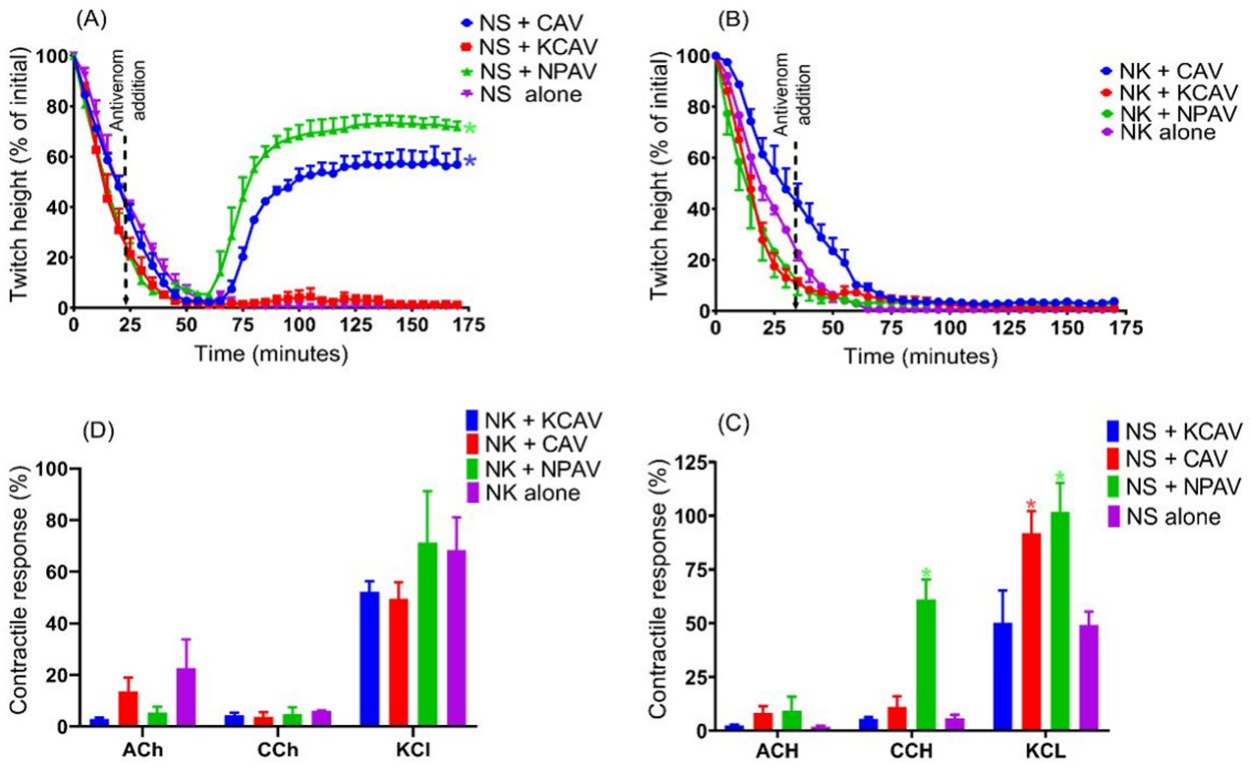
The effect of the addition of antivenom at the t_90_ time point after exposure to Malaysian *N*. *sumatrana* venom and *N*. *kaouthia v*enom to the indirectly stimulated twitches and response to exogenous agonists. Panels (A) and (B) venom indicate change of indirect twitches when recommended titer of Cobra Antivenom (CAV), Neuro Polyvalent Antivenom (NPAV) and King Cobra Antivenom (KCAV) was added at t_90_ point after addition of 5 μg/ml *N*. *sumatrana* venom and *N*. *kaouthia* venom respectively. Panels (C) and (D) venom indicate contractile responses to exogenous agonists in tissues that were added with recommended titer of Cobra Antivenom (CAV), Neuro Polyvalent Antivenom (NPAV) or King Cobra Antivenom (KCAV) at the t_90_ time point after addition of 5 μg/ml *N*. *sumatrana* and *N*. *kaouthia* venoms, respectively. NS indicates *N*.*sumatrana* and NK indicates *N*.*kaouthia* * *p* < 0.05, significantly different from venom alone (n = 3, one-way ANOVA).

Addition of recommended titer of CAV or NPAV at the t_90_ time point, following the addition of *N*. *sumatrana* venom (5 μg/ml) significantly restored the twitch height compared to venom alone i.e., 56.8 ± 6.2% for CAV and 71.6 ± 2.4% for NPAV (Fig 6A). However, the addition of KCAV at the t_90_ time point, following the addition of *N*. *sumatrana* venom, had no significant effect (Fig 6A). Interestingly, the addition any of antivenoms at the t_90_ time point following *N*. *kaouthia* venom (5μg/ml) failed to restore the twitch height (Fig 6B). Contractile responses to CCh and KCl were significantly restored after the addition of NPAV following *N*. *sumatrana* venom i.e., 61.1 ± 9.2% and 101.7 ± 13.6%, respectively. Only contractile response to KCl was restored after addition of CAV (Fig 6C). None of the antivenoms was able to prevent inhibition of ACh contractile response for either venom (Fig 6C and 6D).

### Myotoxic effect of *N*.*sumatrana* and *N*.*kaouthia* venoms in directly stimulated preparation

**Fig 7:**
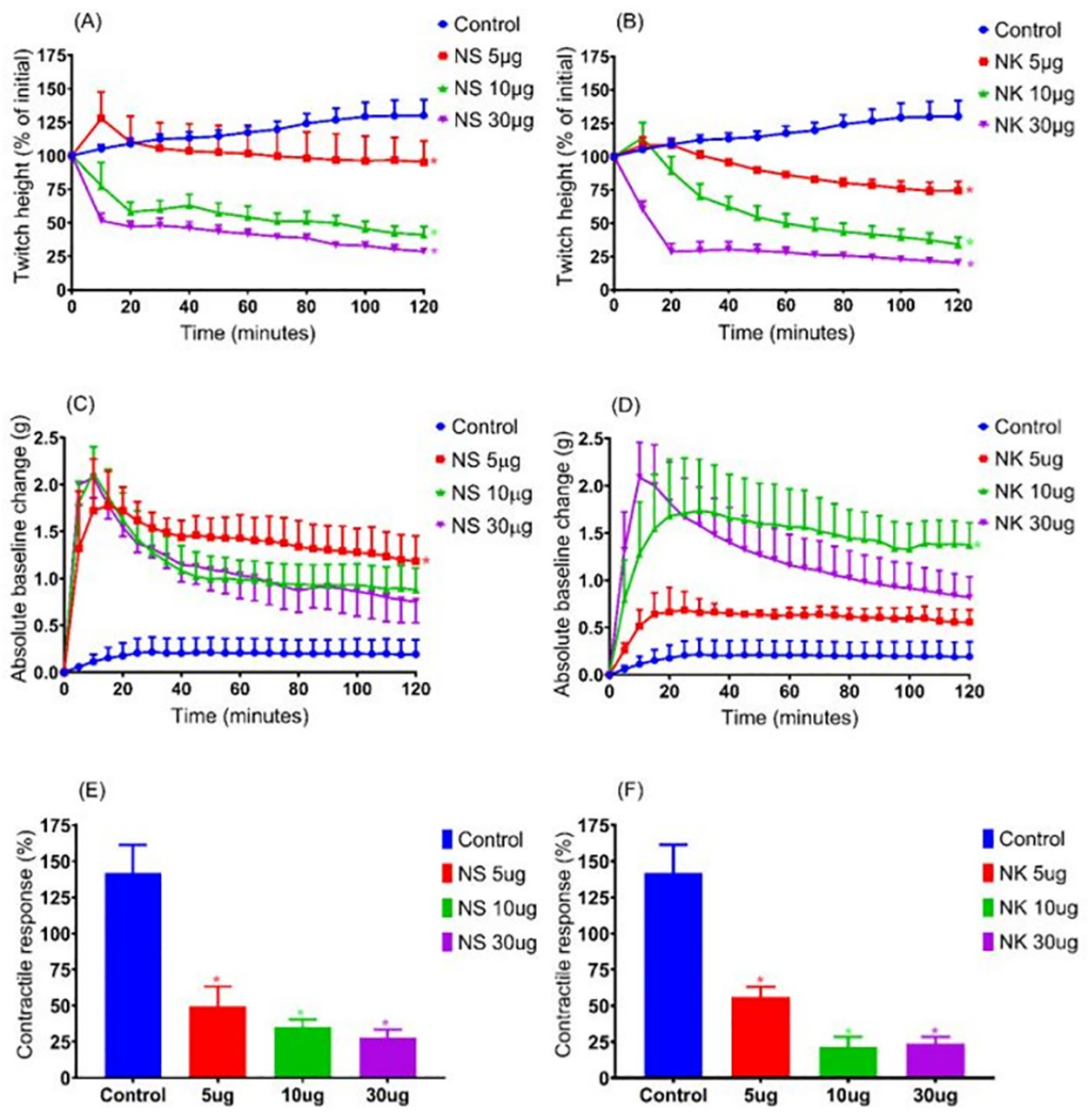
Myotoxic effects of Malaysian *N*. *sumatrana* venom and *N*. *kaouthia* venom in directly stimulated twitches of the chick biventer cervicis nerve-muscle preparation. Panel (A) *N*.*sumatrana* venom and (B) *N*.*kaouthia* venom indicate change in the direct twitch height in tissue added with different concentration (5–30 μg/ml) venom. Panel (C) *N*. *sumatrana* venom and (D) Absolute baseline change of tissues after addition of (C) *N*. *sumatrana* venom and (D) *N*. *kaouthia* venom while on direct twitches. Panel (E) *N*. *sumatrana* venom and (F) *N*. *kaouthia* venom indicate contractile response change to exogenous KCl in tissues exposed to on contractile responses to exogenous KCl. NS indicates *N*.*sumatrana* venom and NK indicates *N*.*kaouthia* venom.* *p* < 0.05, significantly different from control (distilled water) (n = 4, one-way ANOVA).

Both *N*. *sumatrana* and *N*. *kaouthia* venoms (5, 10 and 30 μg/ml) caused concentration-dependent reduction of direct-twitches in the chick biventer cervicis nerve-muscle preparation (Fig 7A and 7B). A significant change of baseline tension was not observed at a venom concentration of 5 μg/ml. The baseline tension for both venoms at 10 and 30 μg/ml significantly increased over the duration of the experiment (Fig 7C and 7B) (n = 4; one- way ANOVA, *p* < 0.05). Both venoms significantly reduced the contractile response to exogenous KCl (Fig 7E and 7F).

### Myotoxicity prevention by antivenom pre-incubation

**Fig 8:**
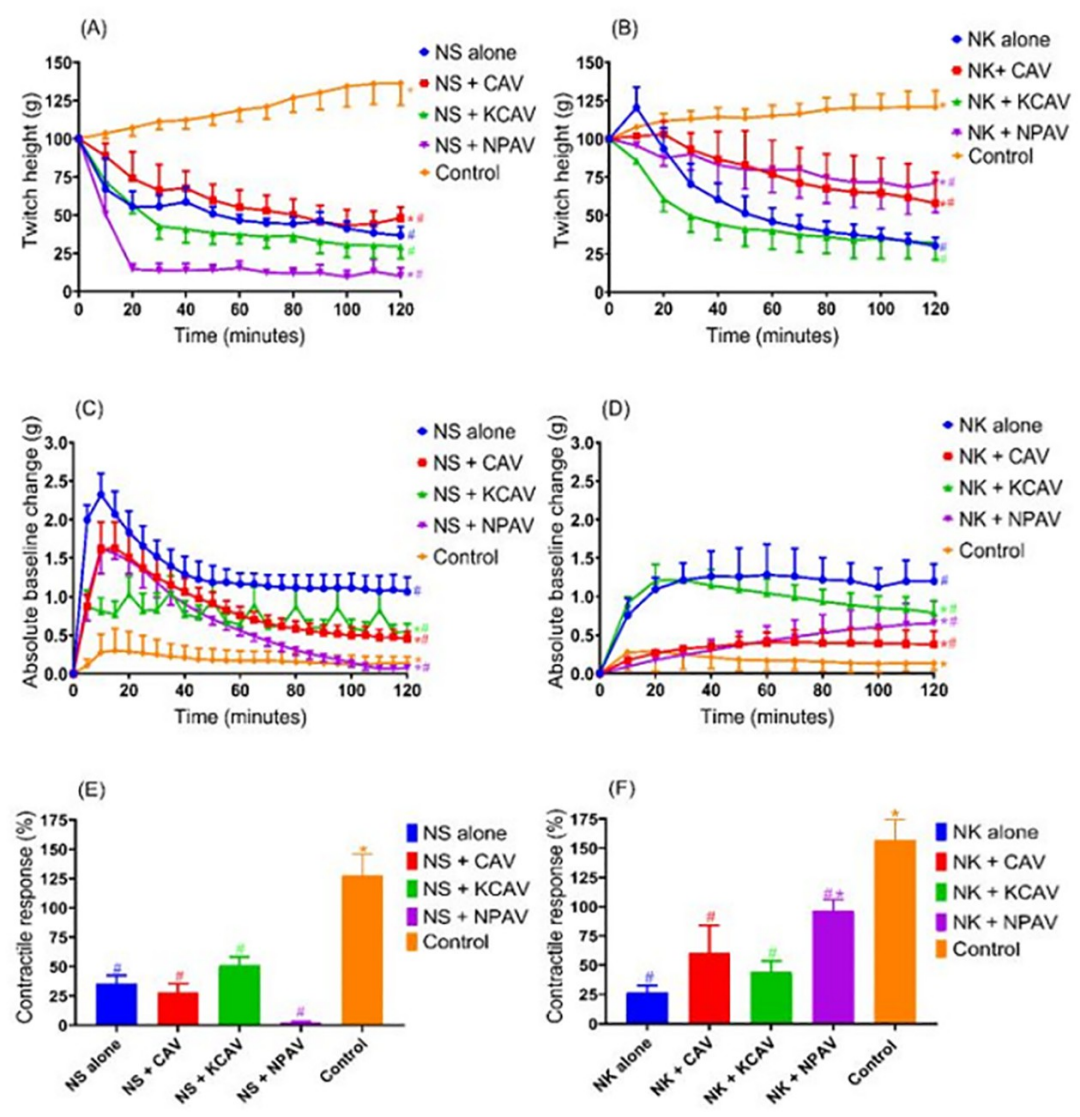
Effect of pre-incubation with antivenom on directly stimulated twitches of tissues and exposed to Malaysian *N*.*sumatrana* and *N*.*kaouthia*. Panel (A) *N*. *sumatrana* venom (10 μg/mL) and (B) *N*. *kaouthia* venom (10 μg/mL) indicate change of direct twitch height in tissues with prior addition of recommended titer of Cobra Antivenom (CAV), Neuro Polyvalent Antivenom (NPAV) and King Cobra Antivenom (KCAV). Panel (C) *N*. *sumatrana* venom and (D) *N*. *kaouthia* venom indicate absolute baseline change of tissues with prior antivenom addition and exposure to venom while on direct simulation. Panel (E) *N*. *sumatrana* venom and (F) *N*. *kaouthia* venom indicate change in contractile responses to exogenous KCl in tissue with prior addition of recommended titer Cobra Antivenom (CAV), Neuro Polyvalent Antivenom (NPAV) and King Cobra Antivenom (KCAV). NS indicates *N*.*sumatrana* venom and NK indicates *N*.*kaouthia* venom.* *p* < 0.05, significantly different than the respective venom alone and # *p* < 0.05, significantly different than control (distilled water) (n = 3, one-way ANOVA).

Pre-incubation of directly stimulated tissues with recommended titer of CAV, KCAV or NPAV, prior to the addition of *N*. *sumatrana* venom (10 μg/ml), did not prevent attenuation of direct twitches compared to control (Fig 8A and 8B) or prevent the inhibition of contractile responses to KCl (Fig 8A) caused by the venoms. Only NPAV reduces the increase in baseline tension in tissues exposed to *N*. *sumatrana* (Fig 8A). In contrast, pre-incubation of CAV and NPAV, prior to the addition of *N*. *kaouthia* venom (10 μg/ml), reduced the inhibition of direct twitches (Fig 8B) and significantly reduced the increase in baseline tension caused by the venom (Fig 8B). Only NPAV significantly attenuated the reduction in the contractile response to KCl exposed to *N*. *kaouthia* venom (Fig 8B).

## Discussion

This study examined the *in vitro* neurotoxicity and myotoxicity of *N*. *sumatrana* and *N*. *kaouthia* venoms in a skeletal muscle preparation. Neurotoxicity is an important clinical feature *N*. *sumatrana* and *N*. *kaouthia* envenoming. Many elapid snakes can induce neurotoxicity by two types of toxins, with different sites of action, found in their venoms i.e presynaptic and postsynaptic neurotoxins. More work has been conducted on neurotoxins from *N*. *kaouthia* venom compared to *N*. *sumatrana* venom [20, 25, 26]. In this study, *N*. *sumatrana* and *N*. *kaouthia* venoms caused concentration-dependent inhibition of indirect and direct twitches in the chick biventer cervicis preparation. Contractile responses of exogenous ACh, CCh and KCl were significantly attenuated by both venoms, indicating postsynaptic neurotoxic and myotoxic activities. Based on t_90_ values, *N*. *kaouthia* venom was more neurotoxic than *N*. *sumatrana* venom. This rank order concurs with previous lethality studies using *in vivo* methods [9, 20, 27]. Cytotoxicity of *N*. *sumatrana* and *N*. *kaouthia* venoms has been previously reported using tissue-based, in vivo, and cell-based assays [28–31]. However, information on the myotoxicity caused by *N*. *sumatrana* venom is relatively limited compared to *N*. *kaouthia* venom [32, 33].

Differences in the potency between *N*. *sumatrana* and *N*. *kaouthia* venoms is likely to be due to the different composition, and quantities, of toxins in the venoms. SDS-PAGE and densitogram profiles for *N*. *sumatrana* and *N*. *kaouthia* venoms revealed different protein migration patterns and number of bands. The higher number of bands peaks in SDS-PAGE and densitogram for *N*. kaouthia venom indicate that the venom is relatively more complex than *N*. *sumatrana* venom. A detailed comparative venomic study is required to confirm this finding. In both venoms, the number of detected venom protein bands in non-reduced samples for both samples are higher than reduced samples. The difference in term of number of protein bands between reduced and non-reduced venoms is attributed to the presence of multimeric proteins. Dimeric of α-cobratoxins and PLA_2_ have been reported from *N*. *kaouthia* venom [34, 35] but not *N*. *sumatrana* venom. Venomic analysis of *N*. *sumatrana* and *N*. *kaouthia* venoms showed that three finger toxins and PLA_2_’s are the two main protein families in the venoms [8, 9]. It is worth to note that venomic analysis of *N*. *kaouthia* venom has been more extensive than *N*. *sumatrana* venom. This could be due to the wider distribution of *N*. *kaouthia* and medical importance of this biting species in the localities where it is found. Geographic variations in the composition of *N*. *kaouthia* and *N*.*sumatrana* venoms were reported to have significant impact of the effectiveness of antivenoms [36–38].

Western blot band pattern and densitogram against the antivenoms used in the current study showed significant differences in the immunoreactivity of antivenoms towards different venom proteins. Cross recognition of antivenoms towards certain venom proteins indicates the homologous nature of some of proteins. Western blot band pattern and densitogram profiles for *N*. *sumatrana* and *N*. *kaouthia* venoms against NPAV and CAV were found to be identical, with a different number of peaks in both venoms. This is not surprising as it is most likely that the same venom source for the production of CAV and NPAV in the same facility. The number of bands and densitogram peaks in KCAV-treated blot are relatively less than in blots treated with CAV and NPAV. Incapability of KCAV in recognizing *N*. *kaouthia* venom proteins in western blot is likely to be due to interspecies variation in venom toxin composition between *N*. *kaouthia* and king cobra (*Ophiophagus hannah*) venoms [39, 40]. This finding is expected as the king cobra is classified in a different genus, and it was recently reported that there could be multiple species of king cobra [41]. Densitogram of *N*. *sumatrana* venom blots that were incubated with NPAV and CAV showed a similar number of peaks, which indicates similar toxin-antibody binding capabilities. However, the number of detected peaks in the blots is lower that the number of peaks from SDS-PAGE. This indicates that there are *N*. *sumatrana* venom proteins that were not recognized by any of the antivenoms. Unlike *N*. *sumatrana* venom, the number of detected peaks in *N*. *kaouthia* venom blots were higher than the number of peaks in the densitogram for SDS-PAGE. This indicates that NPAV and CAV are selective and are highly immunoreactive towards proteins in *N*. *kaouthia* venom. However, the blots were made by using reduced venoms which changed the original protein structure of proteins in the venoms. It is possible that some venom proteins were not detected or over detected by the antivenom due to alteration of epitope in the protein structure.

Tissue pre-incubation with CAV and NPAV at recommended titer prior to addition of *N*. *sumatrana* venom caused partial attenuation of the twitch blockade whereas pre-incubation with CAV and NPAV prior to addition of *N*. *kaouthia* venom prevented twitch reduction. These results are comparable with previous research that suggested CAV and NPAV shown cross-neutralization with *N*. *sumatrana* and *N*. *kaouthia* venoms [20, 42, 43]. Partial attenuation of indirectly stimulated twitch inhibition by KCAV, despite an apparent lack of immunoreactivity in Western blots, indicates the antivenom was able to recognize and bind to some neurotoxins in the venoms. Three finger toxin and phospholipase A_2_ families are the main toxin families in king cobra venom [39, 40]. These toxins families share similarity in their structural sequence and arrangement, and this allows cross-reactivity with KCAV which was developed against king cobra venom.

Addition of recommended titer of CAV or NPAV, at the t_90_ time point, following *N*. *sumatrana* venom, partially reversed the twitch inhibition. However, no reversal was observed when antivenom was added at the same time point after *N*. *kaouthia* venom. This finding concurs with a previous study that showed the inability of CAV to reverse twitch inhibition caused by *N*. *kaouthia* venom when added at t_90_ [44]. This also showed that the antivenoms were able to recognize, bind and displace neurotoxin in *N*. *sumatrana* venom. Some postsynaptic toxins in the *N*. *kaouthia* venom could bind to the skeletal muscle nicotinic acetylcholine receptor in pseudo-irreversible manner, as seen for previously isolated postsynaptic neurotoxins [45–47]. It is also possible that neurotoxicity of *N*. *kaouthia* venom is partially caused by presynaptic neurotoxins which exert their effect by impairing exocytosis of acetylcholine from the presynaptic terminal [48]. These toxins are often resistant to antivenoms unless the antivenom is administered rapidly. Attenuation of contractile responses to exogenous CCh and KCl for *N*.*sumatrana* were partially reversed after addition of NPAV antivenom but only KCl response was improved after addition of CAV. This showed that NPAV have better toxin recognition and neurotoxic reversal capability than CAV for *N*. *sumatrana* venom. Unlike *N*.*sumatrana*, attenuation of contractile response to ACh and CCh by *N*.*kaouthia* was not reversed after addition of KCAV, CAV or NPAV. Incapability of the antivenoms to restore twitch responses and contractile responses to agonists indicate that all antivenoms were not able to effciently displace *N*.*kaouthia* toxins from postsynaptic receptors. *N*.*kaouthia* venom has been reported to have postsynaptic neurotoxins that are known to display ‘pseudo-irrevesible binding’ [44].

In the myotoxicity prevention studies, pre-incubation with CAV and NPAV reduced twitch attenuation induced by *N*. *kaouthia* venom. However, pre-incubation with any of the antivenoms at their recommended titer failed to prevent the twitch reduction induced by *N*. *sumatrana* venom. This indicates that the antivenoms are not effective in neutralizing the myotoxins in *N*. *sumatrana* venom. It is possible that the antibodies against the myotoxins in the antivenoms are insufficient to completely bind all myotoxins and the use of higher titer antivenom may reverse the myotoxic effect [30, 49]. However, we were unable to conduct experiments using higher titer due to limited availability of antivenom samples. Higher titer and earlier antivenom addition have been shown to give better neutralization effects for *N*. *kaouthia* [44]. The myotoxic effect observed could be due to a direct cytotoxic effect of the venom. Cytotoxins have been reported to be present in *N*. *sumatrana* and *N*. *kaouthia* venoms [29] but there is limited information on their actions in skeletal muscle.

There are several limitations of chick biventer muscle preparation in assessing venom activities. The findings using this preparation may not be similar to the effects observed using mammalian tissue preparation. Toxins in snake venom have been reported to have species specificity [50–52] and this could lead to inaccurate assessment of neurotoxicity and myotoxicity of the assayed venom. In addition, findings from isolated tissue preparation should not be directly applicable in clinical setting due to the limited scope parameter in the experimental design. Ideally, an isolated mammalian tissue preparation e.g., isolated murine skeletal muscle preparation, should be included in the study to observe potential species specificity of the venoms. Unfortunately, the accessories required to perform this experiment were not available in our laboratory during the study period. The advantage of chick biventer preparation compared to other isolated tissue preparation is its ability to differentiate pre-junctional from post-junctional effect of venom. This is due to the presence of focal innervated muscle fiber that mediate electrically stimulated twitch and multiply innervated muscle fiber that can be stimulated by exogenous agonists [53].

## Conclusion

In conclusion, Malaysian *N*. *sumatrana* and *N*. *kaouthia* venoms showed distinguishable neurotoxic and myotoxic activities in the chick biventer cervicis muscle preparation. The difference in the antivenom reversal capability for neurotoxicity indicates unique neurotoxin composition in *N*. *sumatrana* and *N*. *kaouthia* venoms. Incapability of the antivenoms to reverse N. *sumatrana*-induced myotoxicity requires further investigation to determine components that causes the effect. The inclusion of venom or some of neurotoxins from *N*. *kaouthia* and myotoxins from *N*. *sumatrana* as a part of immunogen cocktail used for anti-venom production could enhance neutralization capability of CAV and NPAV.

## Supporting information

S1 Raw images Original SDS-PAGE and western blots pictures. (PDF)

S1 File Data for in vitro neurotoxicity of venoms. (XLSX)

S2 File Data for in vitro neurotoxicity prevention and reversal study using antivenom. (XLSX)

S3 File Data for in vitro myotoxicity of venom. (XLSX)

S4 File Data for in vitro myotoxicity prevention study using antivenom. (XLSX)

## Abbreviations

ACh: Acetylcholine
CAV: Thai Red Cross Cobra Antivenom
CCh: Carbachol
KCAV: Thai Red Cross King Cobra Antivenom
KCl: potassium chloride
MNK: Malaysian *Naja kaouthia*
MNS: Malaysian *Naja sumatrana*
NPAV: Thai Red Cross Neuro Polyvalent Antivenom
SDS-PAGE: Sodium Dodecyl Sulphate Polyacrylamide Electrophoresis
