# Supplementary material for: In Vitro neurotoxicity and myotoxicity of Malaysian Naja sumatrana and Naja kaouthia venoms: Neutralization by monovalent and Neuro Polyvalent Antivenoms from Thailand
Source: PLoS One. 2022 Sep 12;17(9):e0274488. doi: 10.1371/journal.pone.0274488 (PMC9467353; doi:10.1371/journal.pone.0274488)
Supplement: S1 Raw images — (PDF) [file pone.0274488.s001.pdf]

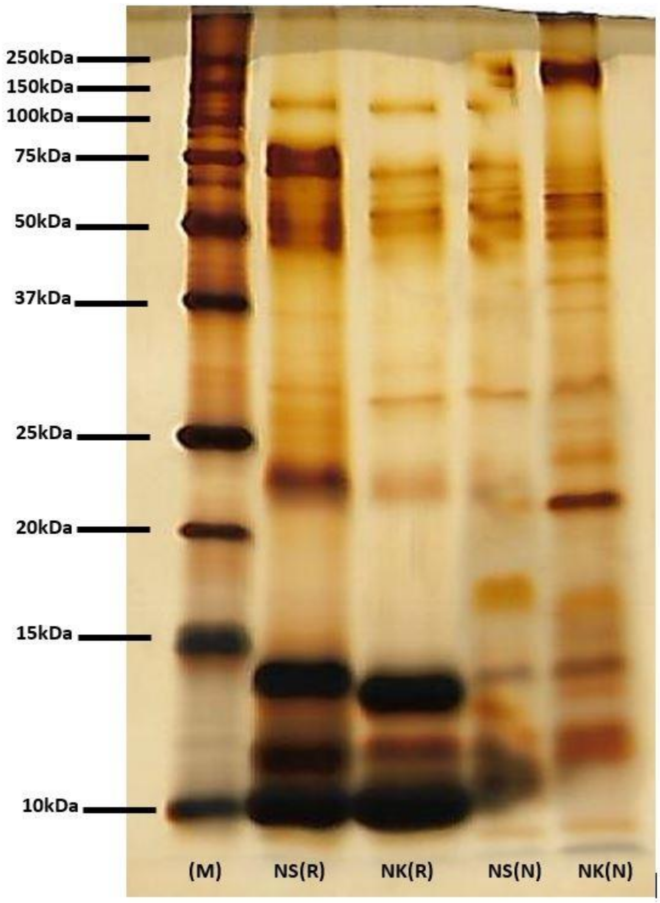

SDS-PAGE of venoms on a 10% gel. Venoms were treated in reducing and non-reducing buffer prior to loading. The gel was stained using silver staining. M indicates the protein marker lane, NS indicates *N. sumatrana* venom, NK indicates *N. kaouthia* venom, (R) indicates venoms treated with reducing sample buffer, (N) indicates venoms treated with non- reducing sample buffer.

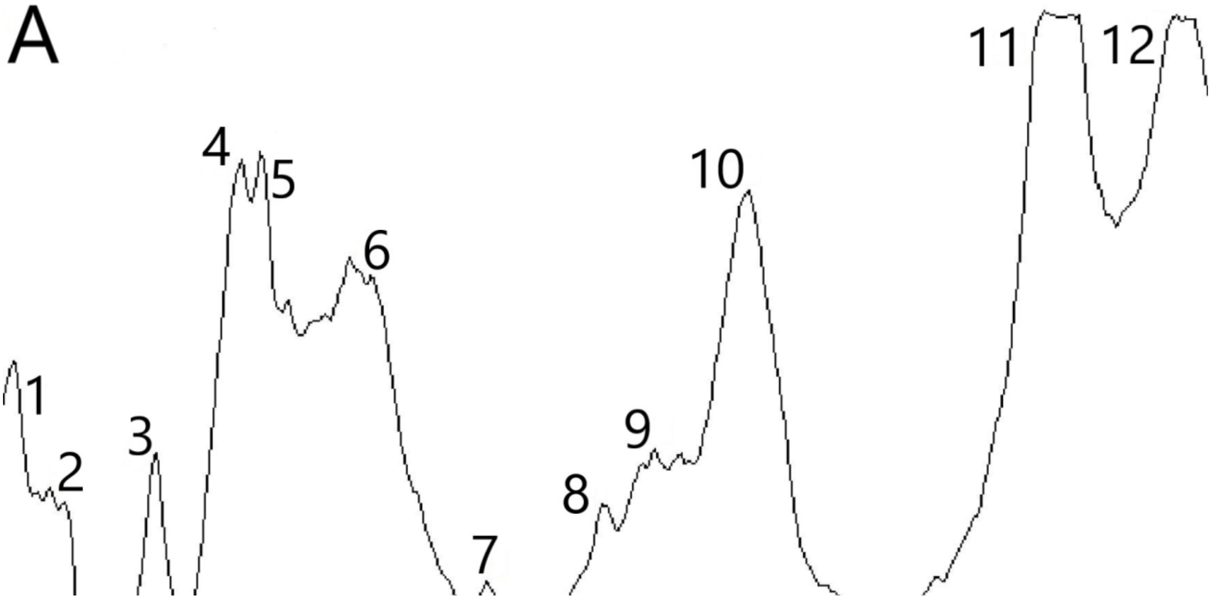

Densitogram for silver-stained SDS-PAGE of Reduced Malaysian *N. sumatrana* venom,

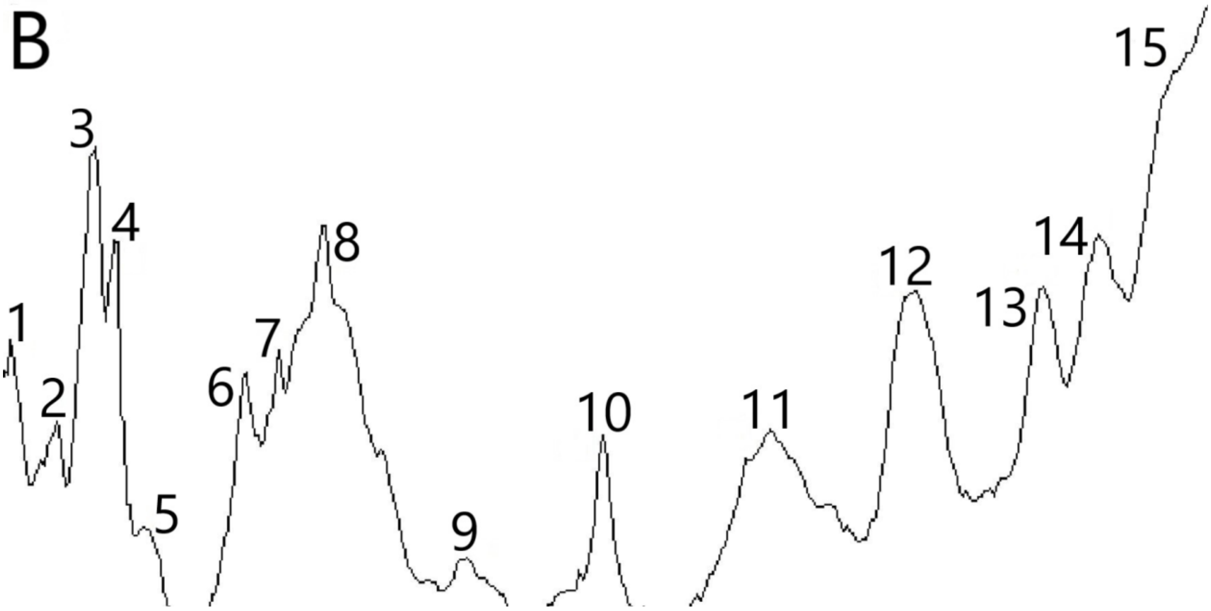

Densitogram for silver-stained SDS-PAGE of Non-reduced Malaysian *N.sumatrana* venom

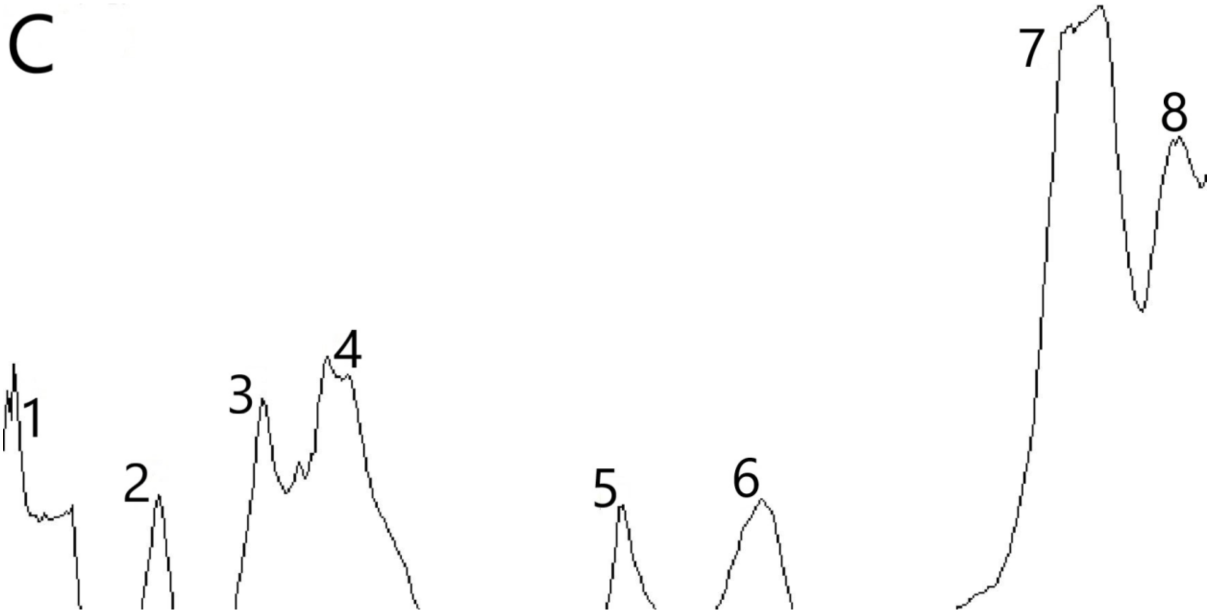

Densitogram for silver-stained SDS-PAGE of Reduced Malaysian *N. kaouthia* venom

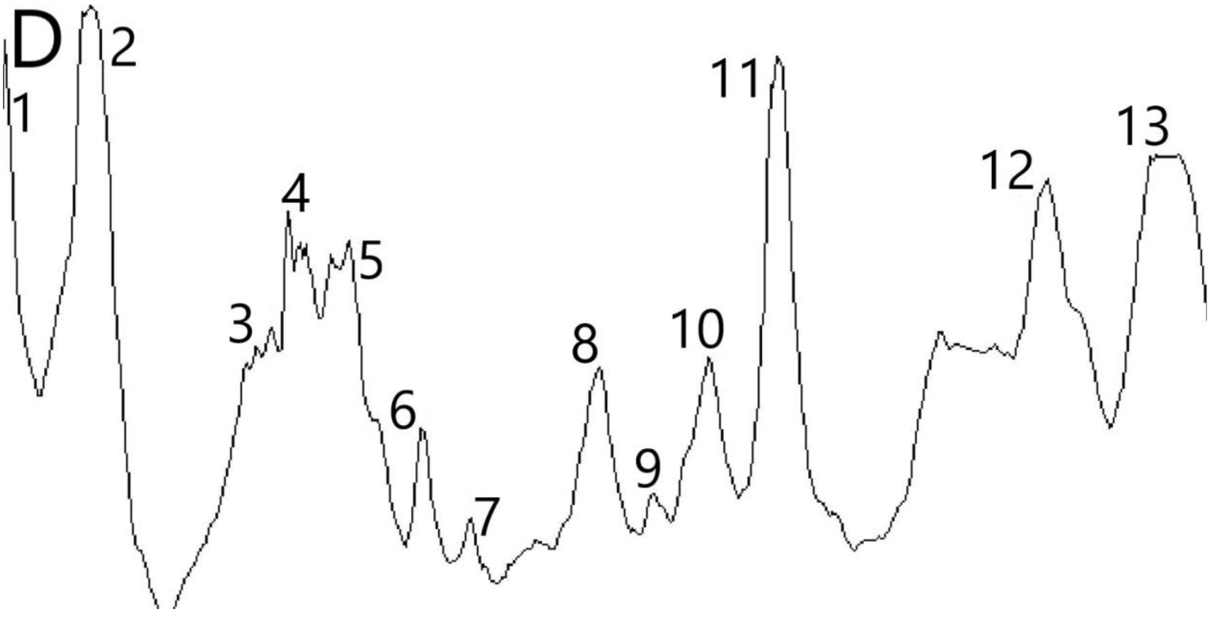

Densitogram for silver-stained SDS-PAGE of Non-reduced Malaysian *N. kaouthia* venom.

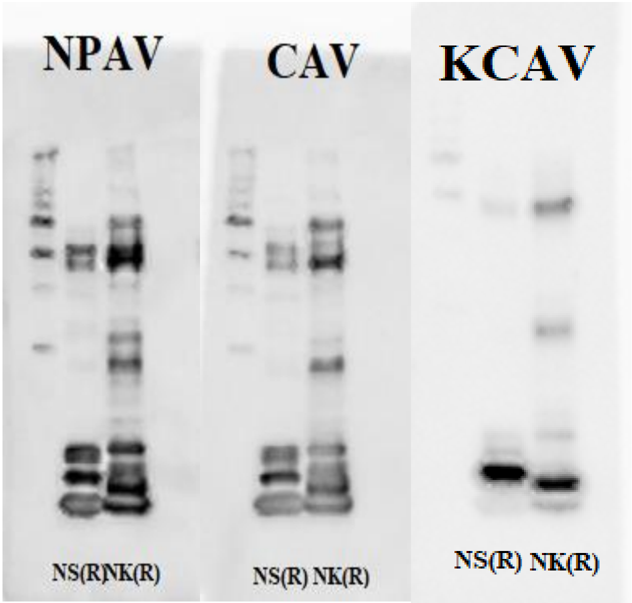

Western blot of reduced *N. sumatrana* and *N. kaouthia* venoms (10 µg). The sample were incubated with Thai Neuro Polyvalent Antivenom, Thai Monocled Cobra Antivenom and Thai King Cobra Antivenom.

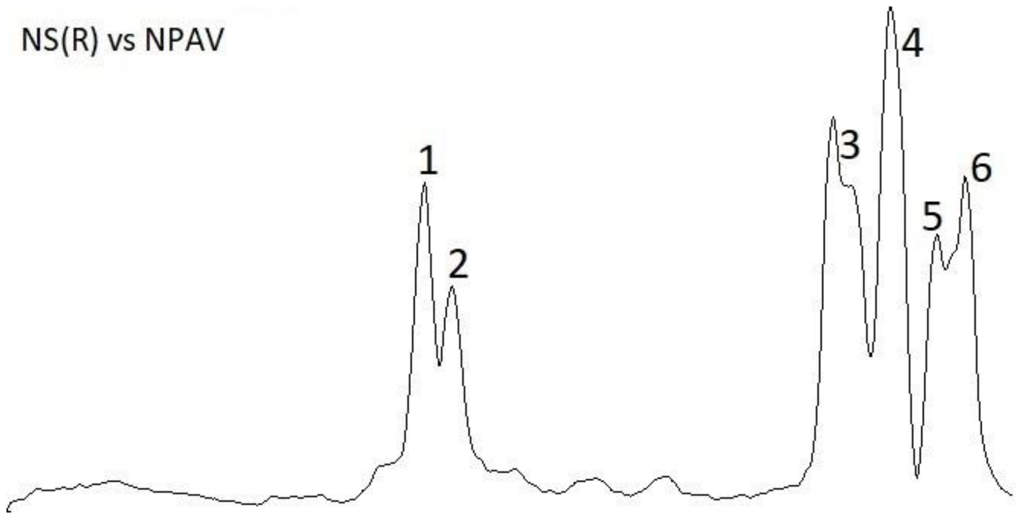

Densitogram for western blot of Malaysian *Naja sumatrana* venom with Thai Neuropolyvalent Antivenom (NPAV),

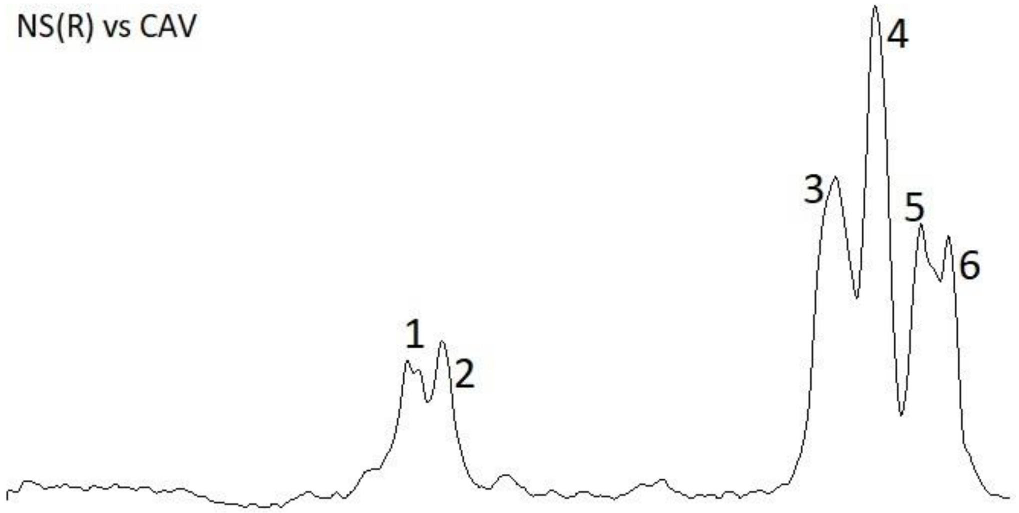

Densitogram for western blot of Malaysian *Naja sumatrana* venom with Thai Monocled Cobra Antivenom (MCAV),

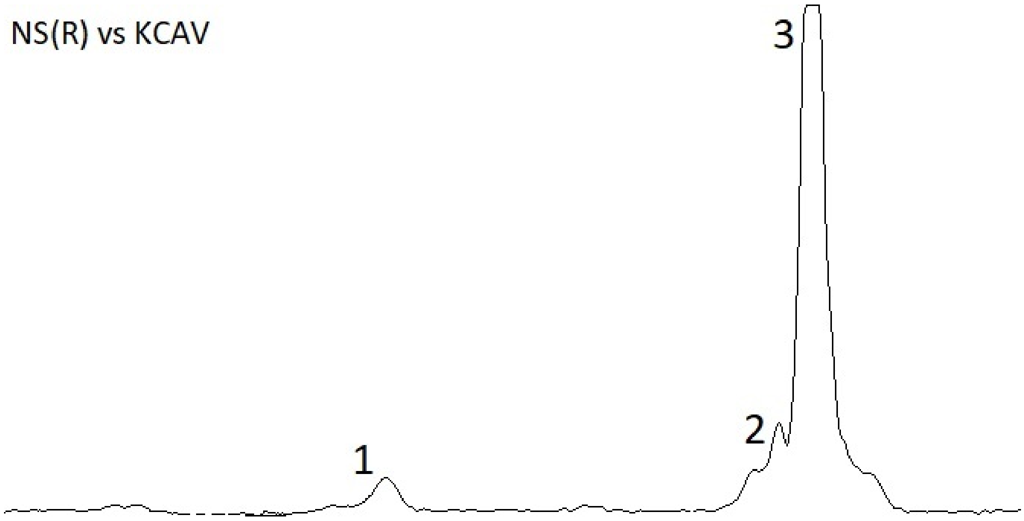

Densitogram for western blot of Malaysian *Naja sumatrana* venom with Thai King Cobra Antivenom (KCAV).

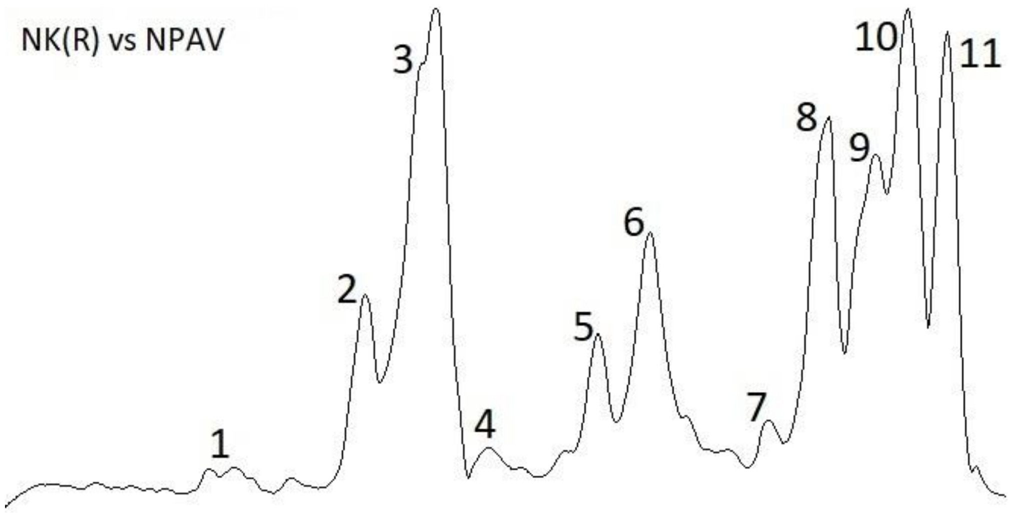

Densitogram for western-blots of Malaysian *Naja kaouthia* venom with Thai Neuropolyvalent Antivenom (NPAV).

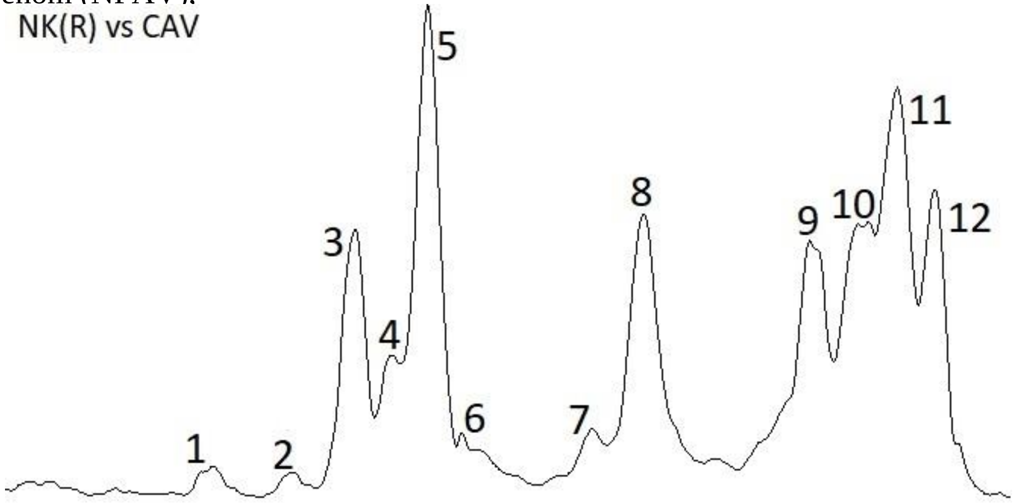

Densitogram for western-blots of Malaysian *Naja kaouthia* venom with Thai Monocled Cobra Antivenom (CAV)

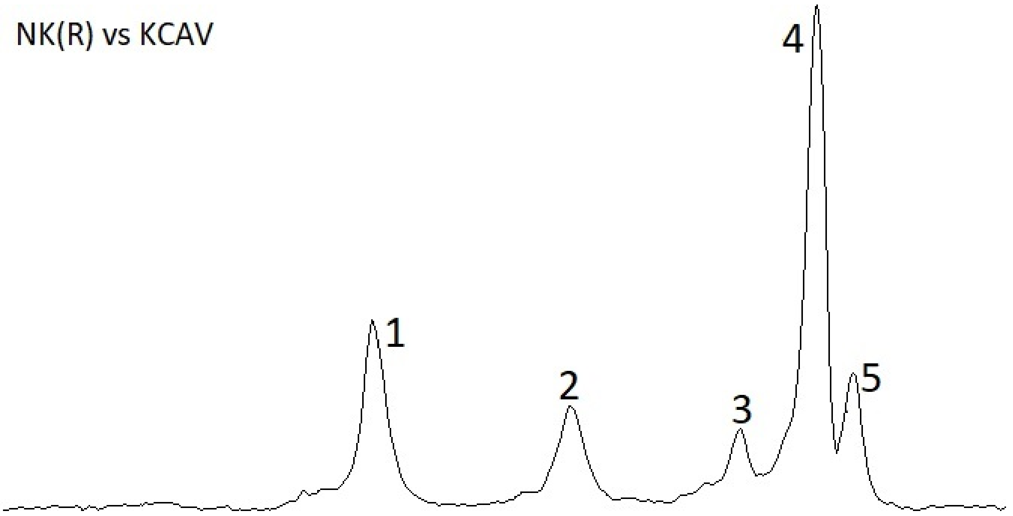

Densitogram for western-blots of Malaysian *Naja kaouthia* venom with Thai King Cobra Antivenom (KCAV).
